# Consecutive Macular Edema and Visual Outcome in Branch Retinal Vein Occlusion

**DOI:** 10.1155/2014/439483

**Published:** 2014-05-22

**Authors:** Sung Uk Baek, Soon Il Kwon, In Won Park, Kyung Jun Choi

**Affiliations:** Department of Ophthalmology, Hallym University Sacred Heart Hospital, No. 896 Pyeongchon-dong, Anyang, Gyeonggi-do 431-070, Republic of Korea

## Abstract

*Purposes*. The study introduced the concept of “consecutive macular edema” and evaluated the validity of visual outcome in macular edema (ME) secondary to branch retinal vein occlusion (BRVO). *Methods*. Patients were categorized into the gainer group and the nongainer group according to the final visual acuity. We analyzed clinical characteristics involving total and consecutive duration of ME between the two groups. *Results*. Among the total 71 eyes of 71 patients, intravitreal bevacizumab injection (26 patients), triamcinolone (21), and natural course (33) were enrolled. The consecutive duration of ME was shorter in the gainer group than in the nongainer group (3.33 ± 1.50 and 5.24 ± 2.39 months; *P* = 0.000). After exclusion of macular ischemia, consecutive duration of ME in gainer group was also significantly shorter than in nongainer group (3.62 ± 1.60 and 6.11 ± 4.20 months; *P* = 0.010). *Conclusions*. The duration of ME in the nongainer group was longer than in the gainer group. In particular, the consecutive duration was an important factor in determining the final visual outcome. *Clinical Trial Registration*. Approval by Hallym University Sacred Heart Hospital Institutional Review Board/Ethics Committee was obtained for this retrospective study.

## 1. Introduction

Branch retinal vein occlusion (BRVO) is the second most common retinal vascular disorder. Decreased visual acuity in BRVO is due to intraretinal hemorrhage and macular edema (ME), capillary nonperfusion, and vitreous hemorrhage from new vessels. ME is the most common cause of decreased visual acuity of BRVO patients. One study reported that 60% of BRVO patients experience ME [1].

With the introduction of many treatments, many studies have assessed visual outcome in ME patients. Prognosis factors of good visual outcome include early improvement of visual acuity [2], subretinal fluid checked in optical coherence tomography (OCT) [3], preoperative visual acuity [4, 5], early treatment [6, 7], type of ME [8], and age [9]. The duration of ME is also an important prognosis factor of good visual outcome [5, 10, 11]. The SCORE study reported a worsened visual outcome with increasing duration of ME [5]. Jaissle et al. [4] correlated shorter duration of ME with improvement of the mean best corrected visual acuity (BCVA).

The patterns of ME are diverse after intravitreal bevacizumab and triamcinolone acetonide injection. ME can recur because the half-life of triamcinolone and bevacizumab in the vitreal cavity is approximately 19 days and 10 days, respectively, and because of the short duration (about 3 months) of the effective concentration [12–14]. Two or three treatments within 5-6 months are common due to recurrence of ME.

The duration of ME can display a complex pattern because of the recurrence of ME after injection. However, previous studies in which ME duration referred to the entire duration did not reflect the complex pattern of recurrence of ME after injection. Thus, we herein introduce the concept of “consecutive duration of ME” and use this concept to analyze the visual outcome in ME patients after BRVO.

## 2. Materials and Methods

### 2.1. Study Population

We retrospectively reviewed the medical records of patients with ME due to BRVO checked by fundus photography and fluorescein angiography from January 2006 to December 2011. The study was performed in accordance with the tenets of the Declaration of Helsinki, and written informed consent was obtained from each study participant. The procedures and possible complications were explained to the patients before the initiation of treatment. Inclusion criteria of ME were a central macular thickness (CMT) exceeding 250 *μ*m on OCT and availability for follow-up period for at least 1 year. The exclusion criteria were corneal opacity leading to loss of vision; cataract; media opacity including vitreous hemorrhage, glaucoma, optic nerve disease, and other retina disorders; and prior panretinal photocoagulation therapy for ME (Table 1).

### 2.2. Data Collection

**Figure 1:**
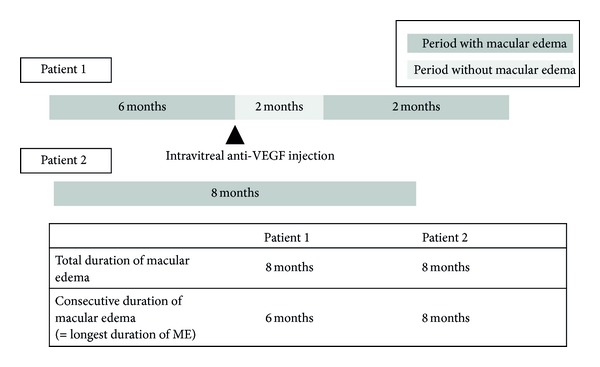
Concept of consecutive duration of macular edema. Patient 1 and patient 2 showed the same total duration of macular edema at the 8-month follow-up. However, different consecutive durations of macular edema were evident (6 months for patient 1 and 8 months for patient 2). VEGF = vascular endothelial growth factor.

Patients were observed at 1, 3, 6, 9, and 12 months from the initial diagnosis day of ME due to BRVO. On ophthalmic exam, BCVA, slit lamp exam, fundus exam, and OCT (Stratus OCT; Carl Zeiss Meditec, Dublin, CA, USA) were checked. Assessment of visual acuity was by the Snellen and converted into LogMAR scale for the analysis. OCT measured the macular thickness around a 1 mm diameter fovea. The thickness of ME was the distance between the internal limiting membrane and the retinal pigment epithelial layer as determined by OCT. BCVA and CMT were measured by OCT in all patients at every clinic visit. The total duration of ME was defined as the sum of the time that CMT exceeded 250 *μ*m regardless of continuity. The consecutive duration of ME was defined as the longest time during which CMT was continuously more than 250 *μ*m. For example, in Figure 1, the duration of ME of two patients was the same as 8 months, but the consecutive duration of ME differed (6 months for patient 1 and 8 months for patient 2). Patients were examined every 3 months that make difficulty in determining the accurate point of ME, such as developed ME and resolved ME. Therefore, when the time point of developed or resolved ME was unclear, we reflected the median time which came up from known exact time of ME. During follow-up, we checked for macular ischemia according to whether foveal capillary bed nonperfusion was observed in fluorescein angiography or not. Ischemic type was defined as capillary bed nonperfusion area exceeding one-third the area of the obstructed vessel area. If we could not determine the perfusion state due to severe retinal hemorrhage, the patient was classified as undetermined.

### 2.3. Treatment

In our clinic, patients with severe ME (>400 *μ*m) that could not be treated by grid laser photocoagulation therapy or patients with massive retinal hemorrhage (more than six disc diameters) were injected with triamcinolone acetonide or bevacizumab. In our experience, ME was treated initially with bevacizumab, particularly in phakic patients, and with triamcinolone acetonide, particularly in pseudophakic patients without glaucoma. Patients who refused any treatment were classified as natural course. If there was no improvement of BCVA, or a worsening of two or more lines, sustained ME, or recurred ME on OCT in patients who had received an intravitreal injection, retreatment was considered. Intravitreal injection of triamcionolone acetonide (Triam, 40 mg/mL/vial; Shin Poong Pharmaceuticals, Seoul, Korea) was given on an outpatient basis in an operating room. The eye received the topical anesthesic proparacaine hydrochloride (Alcaine; Alcon, Fort Worth, TX, USA) and disinfection with 5% povidone iodine. Triamcionolone (4 mg/0.1 mL) was injected using a 30-gauge needle 4 mm posterior to the inferotemporal side of the limbus. Injection of bevacizumab (Avastin, 1.25 mg/0.05 mL; Genetech, San Francisco, CA, USA) was done similarly. Anterior paracentesis was not performed. After injection, topical antibiotics (Levofloxacin and Cravit; Santen, Osaka, Japan) were prescribed.

### 2.4. Outcome Measures

According to the final visual acuity, the gainer group consisted of eyes with a gain of 0.2 or more in LogMAR chart and the nongainer group consisted of eyes with less than 0.2 improvement in LogMAR or which had worsened at the last follow-up visit. Comparative clinical characteristics such as age, initial BCVA, initial CMT, duration of symptom, total duration of ME, and consecutive duration of ME were analyzed between the two groups. We checked underlying disease such as diabetes, hypertension, or cardiovascular disease with a medical examination by interview at initial visit.

### 2.5. Statistical Analysis

SPSS software version 12.0 (SPSS, Chicago, IL, USA) was used for all analyses. A *P* value < 0.05 was considered statistically significant. Comparison between gainer group and nongainer group was done by independent *t*-test. Comparison of three groups was done by analysis of variance (ANOVA) test.

## 3. Results

Eighty patients were enrolled. Thirty-seven patients were male and 43 patients were female. The average age of total patients was 59.30 ± 10.82 years, initial BCVA was 0.37 ± 0.27, initial CMT was 539.45 ± 173.4 *μ*m, and the average period of observation was 19.43 ± 8.1 months (Table 2). Only intravitreal bevacizumab injections were administered to 26 patients, 21 patients received only intravitreal triamcinolone acetonide injection, and the natural course group comprised 33 patients. Mean number of injections was 2.21 ± 0.42 for intravitreal bevacizumab and 2.56 ± 0.78 for intravitreal triamcinolone for each group. Comparisons of the three groups concerning age, sex, initial BCVA, initial CMT, duration of follow-up, and number of injections did not reveal significant differences (Table 3).

The gainer group comprised 45 patients and the nongainer group comprised 35 patients. In the natural course group of patients, the gainer group comprised 23 patients and the nongainer group comprised 10 patients. Intravitreal bevacizumab injection was given to 14 patients in the gainer group and 12 patients in the nongainer group. Triamcinolone injection was given to eight patients in the gainer group and 13 patients in the nongainer group (Table 3).

**Figure 2:**
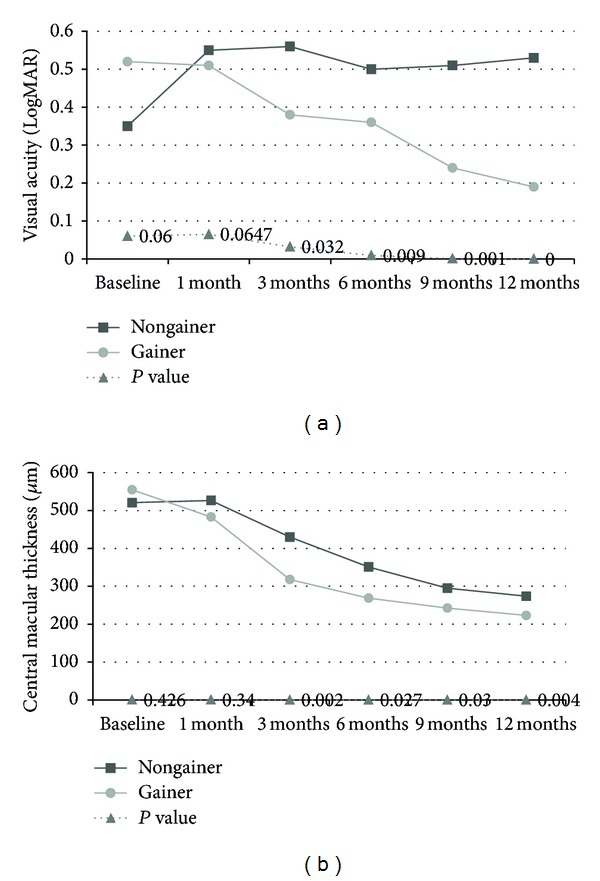
The change of Visual acuity and central macular thickness. (a) Visual acuity and (b) central macular thickness.

BCVA and CMT determined at the initial visit and at 1, 3, 6, 9, and 12 months classified patients in the gainer or nongainer groups (Figure 2). In the gainer group, BCVA gradually increased, while it remained the same after the 1-month follow-up in the nongainer group. A clinically significant difference of BCVA between the two groups (*P* = 0.03) was evident after 3 months (Figure 2(a)). But CMT gradually and similarly decreased in both groups until the 12-month follow-up. Clinically significant difference of CMT between two groups was also evident after 3 months (Figure 2(b), *P* = 0.02).

Related factors between the gainer group and the nongainer group were analyzed (Table 4). There were no significant differences between two groups in age, duration of symptom, initial BCVA, initial CMT, and duration of follow-up. Total duration of ME was longer in the nongainer group (6.87 ± 4.04 months) than in the gainer group (5.23 ± 2.29 months). The difference was significant value (*P* = 0.031). Concerning consecutive duration of ME, the nongainer group (5.24 ± 2.39 months) was significantly longer than the gainer group (3.33 ± 1.50 months) (*P* = 0.00). Consecutive duration of ME had a statistically more significant correlation between two groups than the value of total duration of ME (*P* = 0.031).

Regardless of duration of ME, ischemia of the macula can change the visual outcome. Thus, the perfusion state of the macula can be a disturbing factor. After exclusion of 13 patients with macular ischemia and 18 patients undetermined concerning macular ischemia, reanalysis was performed according to the gainer and nongainer groups (Table 5). As before the exclusion of macular ischemic patients, on analyzing of total duration of ME, the nongainer group (7.29 ± 4.69 months) was longer than the gainer group (5.66 ± 2.59 months), and the difference was significant (*P* = 0.035). Also, like before the exclusion of macular ischemic patients, consecutive duration of ME in the nongainer group (6.11 ± 4.20 months) was also longer than the gainer group (3.62 ± 1.60 months). The difference was clinically significant (*P* = 0.010). Also like before the exclusion of macular ischemic patients, consecutive duration of ME between gainer and nongainer (*P* = 0.010) was statistically more significant correlation than the value of total duration of ME (*P* = 0.035) in good macular perfusion state.

## 4. Discussion

Intravitreal injections of triamcinolone acetonide, bevacizumab, and sustained-release steroid implant other than grid laser photocoagulation are treatments for ME secondary to BRVO. In addition to age, perfusion state of macula, initial treatment time, and response of the first treatment [2–11], the duration of ME is an important prognostic factor for visual outcome [5, 10, 11]. The shorter the duration of ME, the better the visual outcome and prognosis. Intravitreal injection has decreased the duration of ME; however, it is limited by the relatively short-term duration of treatment and the recurrence of ME due to the short half-life of triamcinolone acetonide and bevacizumab. The duration of ME can be diverse and complex.

Patients were categorized into gainer or nongainer groups regardless of the treatments for the analysis of the duration of ME and prognosis of visual outcome. The two groups displayed significant differences in BCVA and CMT after 3 months of follow-up. The visual improvement was consistently associated with decreased CMT in the gainer group, but there was no visual improvement despite the decrease of CMT in the nongainer group. Therefore, no correspondence was evident between functional and structural recovery in the nongainer group. It is conceivable that factors other than CMT are determinants of the final visual outcome. The nongainer group had a longer total duration of ME, so there was a secondary constitutional change of photoreceptors and hence no visual improvement in spite of recovery of macular edema. According to Table 3, untreated eyes (natural course) did better than treated eye. We suggest that natural course tended to involve patients who had good macular perfusion state (data not shown). So, the natural course produced a good visual outcome and had a greater portion of visual gainers than treated eyes.

Previous studies have reported that, in terms of visual outcome, younger age [9], thinner initial CMT [8], and better initial visual acuity are better prognostic factors. However, there was no relationship between these factors and visual outcome in the present study. The only important factors were the total duration of ME and the consecutive duration of ME. The finding that the consecutive duration of ME was a more statistically significant prognostic factor of visual outcome than total duration of ME was a noteworthy finding. Other studies reported that ME due to BRVO leads to functional abnormality with optic nerve cell's extinction [15] and that irreversible visual damage occurs due to the damage of photoreceptor in chronic ME [16]. The present findings support the view that persistent ME gives rise to increased structural damage of photoreceptors. Appropriate timing of treatment is required for reducing the consecutive duration of ME.

There are many confounding factors between visual outcome and the duration of ME. Macular ischemia affects visual outcome, irrespective of the duration of ME [17]. The visual prognosis of patients with macular edema who are treated with intravitreal bevacizumab is influenced by the presence of retinal ischemia [18, 19]. In our study, the nongainer group showed an increased frequency of macular ischemia. In this respect, we compared the duration of ME and the consecutive duration of ME between the gainer group and the nongainer group, except in the case of the macula ischemia patients and the patients with unconfirmed perfusion state. The gainer group showed short duration of ME and consecutive duration of ME, which echoed the result obtained before exclusion of cases of macula ischemia. The consecutive duration of ME was more statistically significant than the total duration of ME. Characteristically, the duration of ME showed a more distinct difference between the gainer group and the nongainer group after analysis, excluding cases of macula ischemia (Tables 4 and 5).

We conclude that the total duration of ME and the consecutive duration of ME are the prognostic factors of visual outcome of patients with ME secondary to BRVO. In particular, it is more important to decrease the consecutive duration of ME. The effectiveness of intravitreal triamcinolone acetonide injection and intravitreal bevacizumab injection was low due to the tendency of ME recurrence after the treatments. Although the total duration of ME was not different because of recurrence, a single intravitreal injection in appropriate time was important to decrease the consecutive duration of ME. Therefore, the use of intravitreal bevacizumab as a rescue is meaningful and beneficially affects visual outcome during long-term observation.

The study was limited because of its retrospective design and the small number of cases. Since the periods of the first injection of triamcinolone acetonide or bevacizumab were manifold, the acute phase patients and the chronic phase patients were blended. Due to the retrospective design and long interval of follow-up, it was not accurate to reflect the exact duration of ME. Further long-term evaluations and a prospective study with many cases are recommended.

## Figures and Tables

**Table 1:** Inclusion and exclusion criteria of patients in this study.

Inclusion criteria	
Central involved macular edema secondary to BRVO	
OCT, central macular thickness > 250 *μ*m	
Patients who were available for a follow-up period of at least 1 year	

Exclusion criteria	
Best corrected visual acuity (LogMAR) > 0.1	
Any new vessel formation	
Other diseases that affect central vision—corneal opacity, cataract, other macular diseases, glaucoma, and so forth.	
Previous grid laser treatment	

BRVO: branch retinal vein occlusion; OCT: optical coherence tomography.

**Table 2:** Baseline characteristics of total subjects.

Demographic data of total patients	Variables
Number of eyes	80
Gender (M : F)	37 : 43
Mean patient age (years)*	59.30 ± 10.82
Baseline V/A (logMAR)*	0.59 ± 0.34
Baseline CMT (*μ*m)*	539.45 ± 173.4
Duration of F/U (months)*	19.43 ± 8.1

*Mean ± standard deviation.

**Table 3:** Demographic data for each group.

Characteristic	Natural course	IVB	IVTA	*P*-value
Number of eyes	33	26	21	
Gainer : nongainer (*n*)	23 : 10	14 : 12	8 : 13	
Mean patient age (years)*	57.57 ± 9.06	57.61 ± 12.11	63.7 ± 11.57	0.487
Gender (M : F)	18 : 15	11 : 15	8 : 13	0.153
Baseline V/A (LogMAR)*	0.56 ± 0.41	0.67 ± 0.43	0.55 ± 0.52	0.248
Baseline CMT (*μ*m)*	517.63 ± 175.43	557.66 ± 157.87	559.05 ± 187.35	0.321
Duration of F/U (months)*	19.45 ± 9.15	19.05 ± 7.36	19.75 ± 7.21	0.481
Number of injections*		2.21 ± 0.42	2.56 ± 0.78	0.165

*Mean ± SD; IVB: intravitreal bevacizumab; IVTA: intravitreal triamcinolone acetonide; F/U: follow-up.

**Table 4:** Evaluation of prognostic factors between the 2 groups.

Characteristics	Gainer (45 patients)	Nongainer (35 patients)	*P* value
Age (years)*	58.15 ± 9.69	60.71 ± 12.07	0.324
Duration of symptom (days)*	34.53 ± 71.06	28.31 ± 45.69	0.471
Baseline visual acuity* (logMAR)	0.30 ± 0.20 (0.66 ± 0.37)	0.45 ± 0.33 (0.54 ± 0.50)	0.060
Baseline CMT (*μ*m)*	554.79 ± 152.52	520.75 ± 196.78	0.414
Macular ischemia			
Yes	5 (11.1%)	8 (22.8%)	
No	32 (71.1%)	17 (48.5%)	
Undetermined	8 (17.7%)	10 (28.5%)	
Mean follow-up (months)*	19.76 ± 8.49	19.03 ± 7.71	0.705
Total duration of ME (months)*	5.23 ± 2.29	6.87 ± 4.04	0.031
Consecutive duration of ME (months)*	3.33 ± 1.50	5.24 ± 2.39	0.000

*Mean ± SD; CMT: central macular thickness; ME: macular edema.

**Table 5:** Analysis of duration of ME between 2 groups after exclusion of macular ischemia and undetermined perfusion state.

Except macular ischemia and undetermined perfusion state	Gainer (25 patients)	Nongainer (16 patients)	*P* value
Total duration of ME*	5.66 ± 2.59	7.29 ± 4.69	0.035
Consecutive duration of ME*	3.62 ± 1.6	6.11 ± 4.2	0.010

*Month, mean ± SD; ME: macular edema.
